# Relative Sinus Bradycardia: An Unexpected Finding in Preeclampsia With Acute Pulmonary Edema

**DOI:** 10.7759/cureus.13262

**Published:** 2021-02-10

**Authors:** Natthapon Angsubhakorn, David Benditt

**Affiliations:** 1 Department of Medicine, University of Minnesota, Minneapolis, USA; 2 Cardiac Arrhythmia Center, Cardiovascular Division, University of Minnesota, Minneapolis, USA

**Keywords:** high blood pressure, sinus bradycardia, baroreceptor, preeclampsia

## Abstract

Preeclampsia is a multifactorial pregnancy-specific syndrome, which can result in significant alterations in cardiovascular hemodynamics. We report an observation of unexpected relative bradycardia in a previously healthy woman who presented with postpartum preeclampsia and acute pulmonary edema. We observed an increase in heart rate following normalization of blood pressure, which suggested that the initial slower than expected heart rate may reflect a baroreceptor response to hypertension. Whether this finding should be regarded as a severe aspect of the disease spectrum needs further study.

## Introduction

Preeclampsia is a pregnancy-related condition characterized by new-onset hypertension with proteinuria and/or variable end-organ dysfunction. It is associated with significant alterations in cardiovascular physiology, often including endothelial dysfunction, and may lead to acute pulmonary edema [1-3].

Bradycardia, usually of modest-to-moderate severity given the clinical presentation, has been reported as a rare feature of preeclampsia; its mechanism, however, has generally been considered to be unknown [4,5]. The case reported here is illustrative of the potential for postpartum preeclampsia in an otherwise healthy young woman to present with a seemingly inappropriate relative bradycardia despite acute pulmonary edema.

## Case presentation

A previously healthy 34-year-old female presented to the emergency department (ED) with a three-day history of progressive shortness of breath. She had an uncomplicated spontaneous vaginal delivery four days back. Review of systems was remarkable for bilateral leg swelling, paroxysmal nocturnal dyspnea, orthopnea, and headache for the past one day. She had no significant medical illness in the past, in particular no preeclampsia or heart failure with her two prior pregnancies.

**Figure 1:**
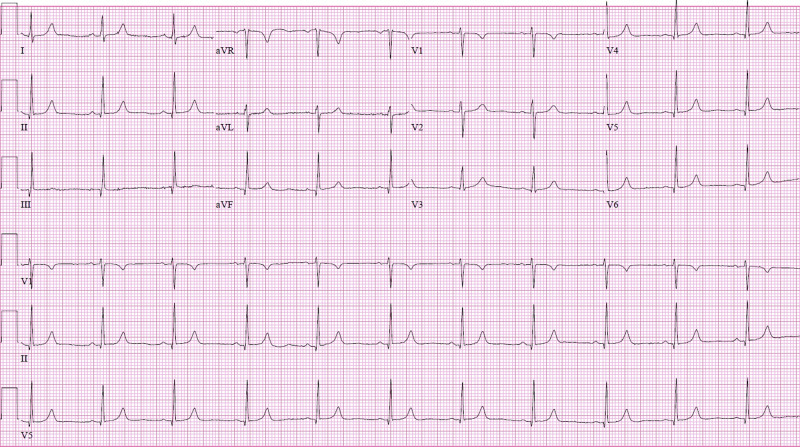
Relative sinus bradycardia despite pulmonary edema Twelve-lead electrocardiogram recorded at the emergency department demonstrating normal sinus rhythm with a relatively slow ventricular rate of 65 bpm and no ST-T segment abnormality despite acute pulmonary edema and moderate oxygen desaturation.

**Figure 2:**
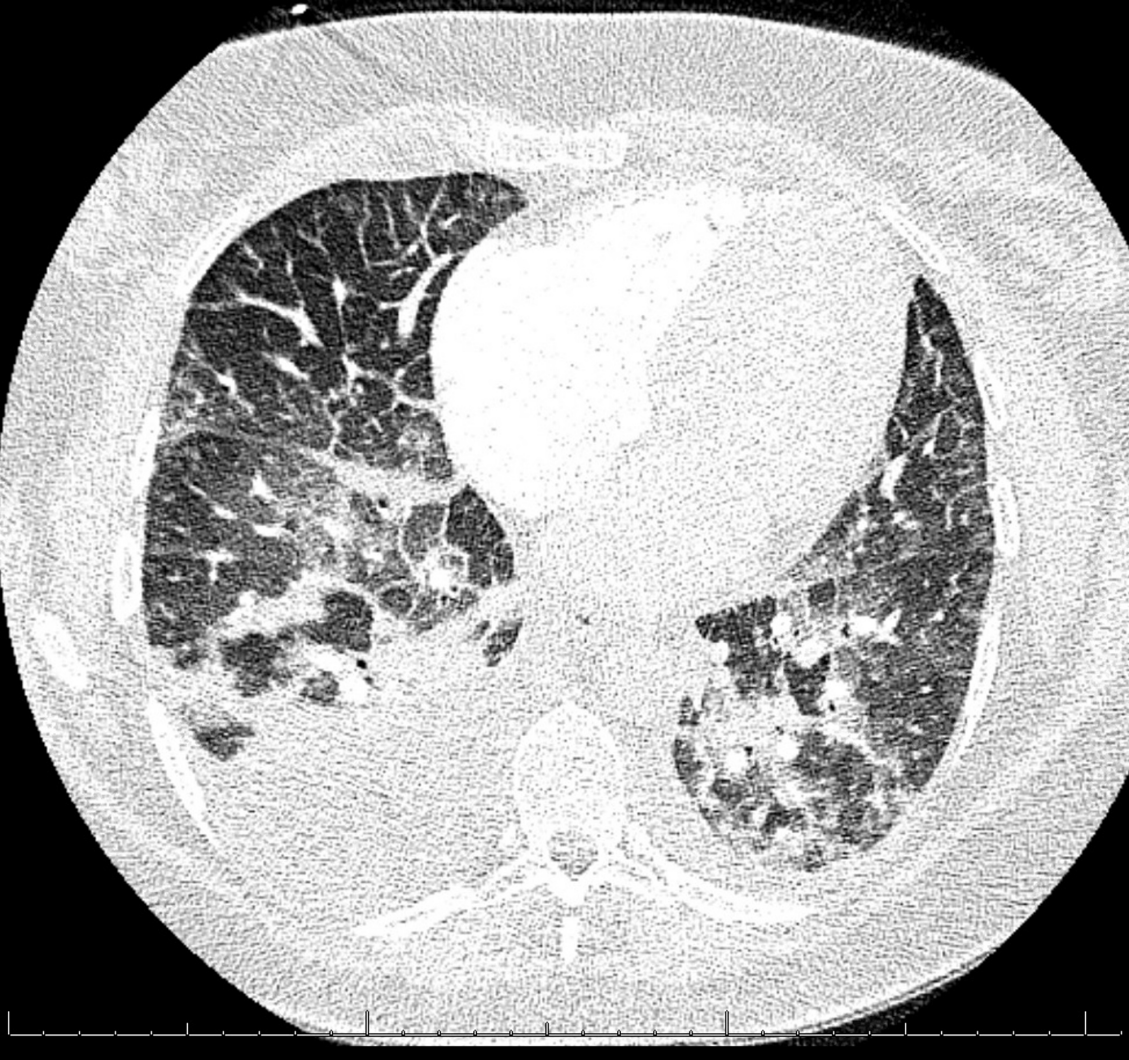
Acute pulmonary edema in preeclampsia Chest CT with contrast showing diffuse bilateral interlobular septal thickening and scattered ground-glass and consolidative opacities suggestive of pulmonary edema; small right and trace left pleural effusion.

In the ED, her temperature was 98.4 °F; respiratory rate, 20 breaths/min; blood pressure, 176/88 mmHg; and pulse rate, 60-64 beats/min. Her initial oxygen saturation was 88% on room air. Physical examination was remarkable for jugular venous distention, fine crackles on chest auscultation, and 2+ symmetrical edema of lower extremities which were warm to the touch. Complete blood count revealed mild anemia but was otherwise unremarkable. The basic metabolic panel was within the normal range. She had low serum albumin of 2.4 g/dL and positive proteinuria. The liver function test was notable for a total bilirubin of 0.1 mg/dL (normal range, 0.2-1.3 mg/dL), alkaline phosphatase of 94 IU/L (normal range, 40-150 IU/L), aspartate aminotransferase 69 IU/L (normal range, 0-45 IU/L), alanine aminotransferase 68 IU/L (normal range, 0-50 IU/L). N-terminal pro-B-type natriuretic peptide (NT-proBNP) was 1,411 pg/mL (normal range, 0 to 450 pg/mL); and troponin I, 0.018 ng/mL (normal range, 0 to 0.045 ng/mL). Electrocardiography demonstrated normal sinus rhythm with a ventricular rate of 65 bpm with no ST-T segment abnormality (Figure 1). Findings on chest CT were consistent with pulmonary edema (Figure 2). A transthoracic echocardiogram (TTE) was obtained and indicated an ejection fraction of 0.55 with an estimated elevated right atrial pressure (RAP) of 15 mmHg and dilated inferior vena cava (IVC) of 2.74 cm. There was no evidence of diastolic dysfunction. She was admitted to the cardiology service for treatment of acute pulmonary edema in the setting of preeclampsia.

Following administration of intravenous furosemide, urine output was 4.9 L within 24 hours, and her respiratory status fully recovered. Intravenous magnesium was given for eclampsia prophylaxis. Her initial hypertension recorded on admission slowly improved from mean arterial pressure of 117 mmHg to 78 mmHg within 14 hours, with diuresis alone. During this period, her heart rate gradually increased from 61 bpm to 90 bpm (Table 1).

**Table 1:** Effect of medical therapy on blood pressure and heart rate profile of a patient with preeclampsia Abbreviation: BP: blood pressure; MAP: mean arterial pressure; HR: heart rate.

	06.00	08.00	10.15	13.30	17.30	20.15
BP (mmHg)	176/88	158/85	153/98		118/74	108/63
MAP (mmHg)	117	109	116		89	78
HR (bpm)	64	64	61		70	90
Medication		Intravenous Magnesium 4 g, then 2 g/h		Intravenous Furosemide 10 mg		

The patient was discharged to home on hospital day 2. Two weeks after the hospitalization, she remained asymptomatic with normal blood pressure and heart rate (104/71 mmHg and 87 bpm, respectively). Her follow-up TTE revealed an ejection fraction of 0.55 with normal estimated RAP and IVC diameter.

## Discussion

The principal observation in this report is the potential for unexpected sinus bradycardia to be a presenting feature of preeclampsia, despite severe associated clinical findings. In this case, the combination of relative sinus bradycardia in conjunction with hypertension and acute pulmonary edema and oxygen desaturation in an otherwise young woman was deemed inappropriate. Potentially, the decreased heart rate was a reflex response to elevated blood pressure, as it normalized with treatment (Table 1) and the patient had no evident underlying cardiac explanation for the observation.

Preeclampsia with severe features can be diagnosed in pregnant women with blood pressure ≥ 160/110 who have at least one of multisystemic signs, including renal dysfunction, elevated serum transaminases, pulmonary edema, thrombocytopenia, and new-onset cerebral or visual disturbances [1]. Acute pulmonary edema occurs in 3% of women with preeclampsia, with 70% of cases occurring during the postpartum period [6]. It can present with respiratory symptoms mimicking other life-threatening cardiopulmonary conditions such as pulmonary embolism, acute coronary syndrome, and peripartum cardiomyopathy. Imaging, particularly echocardiography, is useful for differentiating primary cardiac causes from those secondary to preeclampsia. Inasmuch as the incidence of preeclampsia in multiparous women with previous normal pregnancies is low [7], diagnosing this rare presentation requires a high index of suspicion.

Pathophysiology of preeclampsia

Although the pathophysiology of preeclampsia is not fully understood, it is a complex disorder deemed due to placental insufficiency. It is speculated that the placenta, in response to impaired perfusion, produces proinflammatory molecules that result in maternal endothelial dysfunction [8]. Furthermore, maternal cardiovascular maladaptation and predisposing metabolic factors may also play an important role [9]. Cardiovascular effects of preeclampsia are primarily attributable to increased vasoconstrictor activity resulting in high systemic vascular resistance, hypertension, and end-organ hypoperfusion [1,10]. The recent literature suggests that there are two different hemodynamic patterns in women affected by preeclampsia [9,11]. In cases of preeclampsia that occur earlier during gestation, the typical hemodynamic pattern is high total vascular resistance and low cardiac output, reflecting a more significant cardiac burden. In contrast, the second hemodynamic pattern, which is more frequently seen in late-onset preeclampsia and is similar to our case, is normal-to-high cardiac output, increased intravascular volume, and high total vascular resistance. Recognizing these subtypes and their underlying hemodynamics may be helpful for the rational management of preeclampsia [11].

Preeclampsia is known to cause pulmonary edema as a result of increased intravascular hydrostatic pressure due to elevated blood pressure, decreased plasma oncotic pressure from hypoalbuminemia, increased capillary permeability related to endothelial dysfunction, and left ventricular diastolic dysfunction in the setting of increased cardiac afterload [3,6,9]. Intravenous diuretic is used to promote diuresis and venodilation. Urgent management of severe hypertension, if present, with an intravenous antihypertensive agent is important to reduce the risk of further complications [6].

Bradycardia

**Figure 3:**
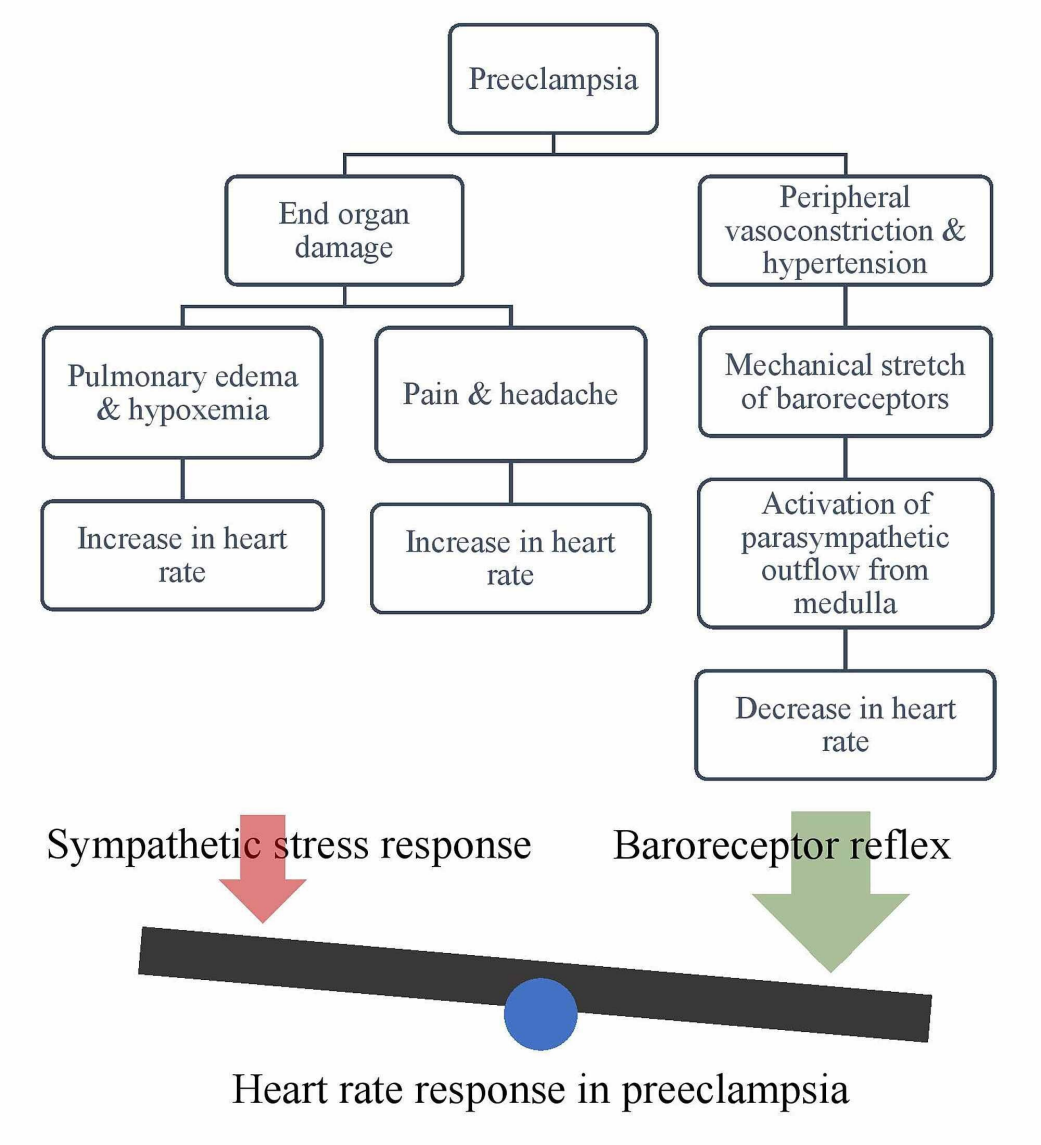
Heart rate response in preeclampsia Diagram illustrating the pathophysiology of preeclampsia, baroreceptor reflex response, and the implications on heart rate. We hypothesize that preeclampsia exerts its effects on heart rate primarily through baroreceptor reflex response to elevated blood pressure, leading to a decrease in heart rate. In addition, preeclampsia may be associated with end-organ damage that can lead to sympathetic stress response and an increase in heart rate.

The mechanism of bradycardia in preeclampsia is unknown [4,5]. Our patient had normal electrolytes and no evidence of acute myocardial ischemia on electrocardiography and TTE. In the absence of an obvious reason for new sinus node dysfunction in this patient, we speculate that a baroreceptor reflex response may be responsible for the development of bradycardia which disappeared after normalization of the blood pressure. In our patient, pulmonary edema, increased RAP, IVC engorgement, diminished oxygen saturation, and elevated NT-proBNP led to a diagnosis of intravascular volume overload. Intuitively, in such a setting, one might expect heart rate to increase in response to anticipated augmented sympathetic activity. However, as noted earlier, the pathogenesis in preeclampsia is mainly related to vasoconstriction, and bradycardia may be the result of a baroreceptor response to severely elevated blood pressure (Figure 3). Our patient’s heart rate increased appropriately following normalization of blood pressure, supporting this hypothesis. We speculate that bradycardia in women diagnosed with preeclampsia may signify a more severe aspect of the disease spectrum, in particular in terms of hypertension severity. Further investigation of this observation in larger cohorts is needed.

## Conclusions

In conclusion, relative bradycardia in the context of preeclampsia, especially given its multiple attendant complications, is unexpected. Its clinical significance is yet to be determined. Our observation suggests that the slower than expected heart rate may reflect a baroreceptor response to hypertension, which becomes apparent only in a subset of affected preeclamptic patients.
